# ATP6V0C Knockdown in Neuroblastoma Cells Alters Autophagy-Lysosome Pathway Function and Metabolism of Proteins that Accumulate in Neurodegenerative Disease

**DOI:** 10.1371/journal.pone.0093257

**Published:** 2014-04-02

**Authors:** Leandra R. Mangieri, Burton J. Mader, Cailin E. Thomas, Charles A. Taylor, Austin M. Luker, Tonia E. Tse, Carrie Huisingh, John J. Shacka

**Affiliations:** 1 Department Pathology, Neuropathology Division, University of Alabama at Birmingham, Birmingham, Alabama, United States of America; 2 Department Ophthalmology, University of Alabama at Birmingham, Birmingham, Alabama, United States of America; 3 Birmingham Veterans Administration Medical Center, Birmingham, Alabama, United States of America; IISER-TVM, India

## Abstract

ATP6V0C is the bafilomycin A1-binding subunit of vacuolar ATPase, an enzyme complex that critically regulates vesicular acidification. We and others have shown previously that bafilomycin A1 regulates cell viability, autophagic flux and metabolism of proteins that accumulate in neurodegenerative disease. To determine the importance of ATP6V0C for autophagy-lysosome pathway function, SH-SY5Y human neuroblastoma cells differentiated to a neuronal phenotype were nucleofected with non-target or ATP6V0C siRNA and following recovery were treated with either vehicle or bafilomycin A1 (0.3–100 nM) for 48 h. ATP6V0C knockdown was validated by quantitative RT-PCR and by a significant decrease in Lysostracker Red staining. ATP6V0C knockdown significantly increased basal levels of microtubule-associated protein light chain 3-II (LC3-II), α-synuclein high molecular weight species and APP C-terminal fragments, and inhibited autophagic flux. Enhanced LC3 and LAMP-1 co-localization following knockdown suggests that autophagic flux was inhibited in part due to lysosomal degradation and not by a block in vesicular fusion. Knockdown of ATP6V0C also sensitized cells to the accumulation of autophagy substrates and a reduction in neurite length following treatment with 1 nM bafilomycin A1, a concentration that did not produce such alterations in non-target control cells. Reduced neurite length and the percentage of propidium iodide-positive dead cells were also significantly greater following treatment with 3 nM bafilomycin A1. Together these results indicate a role for ATP6V0C in maintaining constitutive and stress-induced ALP function, in particular the metabolism of substrates that accumulate in age-related neurodegenerative disease and may contribute to disease pathogenesis.

## Introduction

Vacuolar-ATPase (V-ATPase) is a membrane-associated, multi-subunit protein complex that functions as an ATP-driven proton-pump [1]. V-ATPase is organized into two coordinately operating multi-subunit domains: the peripheral V_1_ domain that performs ATP hydrolysis and the integral V_0_ domain that allows for proton translocation across the membrane. The V_1_ and V_0_ domains are connected to each other by a central “stalk” of shared subunits. The rotary action of the stalk subunits has been proposed to drive proton translocation across the membrane upon V_1_ hydrolysis of ATP.

V-ATPase is localized to many different membranes of eukaryotic cells including lysosomes, endosomes, Golgi-derived vesicles, secretory vesicles and in some cell types the plasma membrane [1]. V-ATPase has well documented functions, including maintenance of both acidic vesicle and cytosolic pH and vesicle fusion with vacuoles [2], [3]. V-ATPase-dependent maintenance of acidic pH in lysosomes and endosomes is important for optimal function of their proteolytic enzymes, whereas V-ATPase-dependent vesicle fusion serves a variety of functions including neurotransmitter release from synaptic vesicles, transport of Golgi-derived lysosomal enzymes and membrane proteins, and effective fusion of autophagosomes with lysosomes and endosomes [1]–[5].

Pharmacologic inhibition of V-ATPase was first reported in 1988 by the use of antibiotic drugs coined “bafilomycins” derived from *Streptomyces* soil bacteria. Bafilomycin A1 and structurally related compounds have in common a 16–18 membered macrolactone ring linked to a unique side chain and together represent the plecomacrolide subclass of macrolide antibiotics [6]. Bafilomycin A1 has been shown to inhibit V-ATPase with high affinity, at concentrations ≥ 10 nM [6]. Bafilomycin A1 and similarly structured compounds are widely used as pharmacologic tools to inhibit lysosome acidification and inhibit autophagy-lysosome pathway (ALP) function by preventing autophagosome-lysosome fusion, thus promoting the robust accumulation of autophagosomes [2], [3], [7]–[9]. It is believed that V-ATPase-dependent vesicle fusion also requires the maintenance of acidic pH, though recent studies have indicated that fusion may occur in a pH-independent manner [10], [11].

It is widely believed that inhibition of ALP function contributes to the aberrant accumulation of protein species in age-related neurodegenerative disease that not only define disease-specific neuropathology but also may contribute to disease pathogenesis, including alpha synuclein (α-syn) in Parkinson's disease and metabolites of amyloid precursor protein (APP) in Alzheimer's disease [12]–[15]. Several ALP-associated molecules have been identified that are currently being investigated for their utility as therapeutic targets in age-related neurodegenerative disease [13], [15]–[23]. Experimental inhibition of V-ATPase by bafilomycin A1 prevents the effective degradation of α-syn that in turn promotes accumulation of α-syn soluble oligomeric and insoluble aggregate species with neurotoxic potential [24]–[29]. Bafilomycin A1-mediated inhibition of V-ATPase also effectively inhibits the rapid degradation of full-length APP and its metabolites, C-terminal fragments (CTFs) that are formed initially upon cleavage of full-length APP by β-secretase [30]–[32]. Subsequent cleavage by γ-secretase can promote the generation of toxic Aβ species, whereas subsequent cleavage by α-secretase or γ-secretase can generate the putatively toxic APP intracellular domain (AICD) [30], [32]. As such, the ALP and putatively V-ATPase represent attractive targets for promoting the metabolism of proteins that contribute to the pathogenesis of age-related neurodegenerative disease.

Through analysis of bafilomycin A1-resistant strains of the fungus *Neurospora crassa* it was discovered that bafilomycin A1 inhibition of V-ATPase activity is mediated by binding with high affinity to the c subunit in the V_0_ domain, or ATP6V0C [33]. Knockdown of ATP6V0C has been shown recently to inhibit vesicular acidification and sensitize cells to stress-induced cell death [34]–[36], while ATP6V0C-deficient mice are embryonic lethal [37]. However, whether ATP6V0C itself is responsible for regulating ALP function, as well as the metabolism of substrates that accumulate in age-related neurodegenerative diseases has not been previously investigated. In the present study we found that knockdown of ATP6V0C in neuronal cells adversely affected ALP function concomitant with accumulation of ALP-associated substrates including α-syn and APP-CTFs, and exacerbated stress-induced cell death. Our findings suggest an important role for ATP6V0C in maintenance of ALP function that may portend relevance to age-related neurodegenerative disease.

## Materials and Methods

### Cell Culture

Naïve SH-SY5Y human neuroblastoma cells (ATCC, CRL-2266) were maintained in T-75 flasks (Corning, 430641) at 37°C and 5% CO_2_ in Minimum Essential Media (Cellgro, 10-010-CV) and F12-K media (ATCC, 30–2004) supplemented with 0.5% sodium pyruvate (Cellgro, 25-000-CI), 0.5% non-essential amino acids (Cellgro, 25-025-CI), 1% penicillin/streptomycin (Invitrogen, 15140-122), and 10% heat-inactivated fetal bovine serum (FBS; Thermo Scientific, SH30109.03). Naïve cells received full media change every two days. Prior to their use in experiments, naïve SH-SY5Y cells were differentiated for seven days to a post-mitotic state in 10% FBS media supplemented with 10 μM retinoic acid (RA; Sigma, R2625). Complete RA-supplemented media was replenished every two days.

### Nucleofection with siRNA

Small-interfering RNA (siRNA) specific for human ATP6V0C was obtained from Thermo Scientific. The Amaxa Nucleofector system (Lonza, VVCA-1003), a modified electroporation system, was utilized to transiently knock down ATP6V0C. First, RA-differentiated SH-SY5Y cells were re-suspended in the Lonza Nucleofector reagent. Next, 400 nM of re-suspended ATP6V0C siRNA (Thermo Scientific, M-017620-02-0010), or non-target siRNA (Thermo Scientific, D-001206-13-05), (in siRNA buffer with 20 mM KCl, 6 mM HEPES-pH 7.5, and 0.2 mM MgCl_2_) was added to the cell suspension. After electroporation, 500 μL of FBS-containing media was immediately added to the cell suspension. Following a 10 min recovery period, nucleofected cells were transferred to T-75 flasks and incubated overnight in 10% FBS differentiation media for 24 h. The next day nucleofected cells were plated for experiments (24 h after nucleofection and 24 h prior to drug treatment) in either eight well glass chamber slides (LabTek, 154941), six well plates (Corning 3516) or 60 mm dishes (Corning, 430166) at a density of 500/mm^2^ in 0% FBS differentiation media containing 2% B-27 supplement (Invitrogen, 17504044). All experimental endpoints ended at 96 h after nucleofection.

### Treatment with Bafilomycin A1

Following nucleofection, recovery and plating for experiments (i.e. 48 h after nucleofection), media was exchanged for fresh 0% FBS differentiation media containing 2% B-27 supplement with either DMSO vehicle (0 nM control) or bafilomycin A1 (0.3–100 nM; AG Scientific, B-1183). Bafilomycin A1 was prepared as a 10 mM stock solution in DMSO (Sigma, D8418-1L) and stored at −20°C. Unless otherwise noted, all bafilomycin A1 treatments were for 48 h, with experiments ending 96 h after initial nucleofection.

### Quantitative Real Time PCR

Cells nucleofected with ATP6V0C siRNA or non-target siRNA were processed for total RNA extraction with the use of TRIzol reagent (Invitrogen, 15596-026). cDNA was generated with oligo (dT) from four μg of RNA using the SuperScript III First Strand Synthesis System (Invitrogen #18080-051). Primers used for detection of ATP6V0C mRNA were ATP6VOC 5′-ATGTCCGAGTCCAAGAGCGGC-3′ and ATP6VOC 5′-CTACTTTGTGGAGAGGATGAG-3′. Primers for actin were 5′-GCTCGTCGTCGACAACGGCTC-3′ and 5′-CAAACATGATCTGGGTCATCTTCTC-3′. Template for cDNA (2 μL) was added along with 1X Fast SYBR Green Master Mix (ABI #4385610) and water to a final volume of 20 μL/well. Assays were performed in triplicate on a Bio-Rad CFX96 instrument using the following conditions: (95°C for three minutes followed by 95°C for 15 seconds and 60°C for one minute for 45 cycles. The comparative cycle threshold (Ct) method (ddCT; [38]) was used to determine expression of ATP6V0C in each sample relative to endogenous actin control, which was then used to determine knockdown in ATP6V0C siRNA samples as a percentage of non-target siRNA control.

### Measurement of Acidic Vesicle pH

After treatment for 48 h with DMSO vehicle, cells plated in 60 mm dishes were incubated with LysoTracker Red (LTR; Life Technologies, L7528; 100 nM final concentration) for 30 min in Locke's buffer (15 mM NaCl, 5.6 mM KCl, 3.6 mM NaHCO_3_, 1.3 mM CaCl_2_, 1 mM MgCl_2_, 10 mM HEPES, 1.009 g/L glucose, pH 7.4). Cells were then harvested, pelleted, and re-suspended in 1 X PBS then passed through a 70 μm nylon cell strainer (BD Falcon, 08-771-2) and into collection tubes to provide a single cell suspension. Cells were kept on ice and protected from light during immediate transport to the Joint UAB Flow Cytometry Facility (Enid Keyser, Director) for analysis. A total of 1 × 10^4^ events were detected in each experimental condition using the BD LSR II flow cytometer (Becton Dickinson). Further analysis was performed using FlowJo software (licensed by UAB) to assess mean fluorescence intensity. LTR fluorescence was also visualized via microscopy in cells plated in eight well glass chamber slides and treated for either 48 h with DMSO vehicle or with 100 nM bafilomycin A1 for the last 3 h of the 48 h time course. Following treatment with LTR, fluorescent images were captured using a Zeiss Axioskop fluorescent microscope (Carl Zeiss Micro-imaging, LLC) at 40X magnification. Transmitted light images were also taken using phase contrast optics.

### Western Blot Analysis

After treatment for 48 h, nucleofected cells in 60 mm dishes were incubated for seven minutes at 37°C with Accutase (Innovative Cell Tech., AT104) to detach cells from their substrate. Whole cell lysates were collected and protein concentrations were determined using BCA protein assay (Fisher Scientific, PI-23227) as previously described [8]. Equal amounts of each protein sample were then electrophoresed using 12% Tris/Glycine SDS-polyacrylamide gels, or for APP CTFs using 16.5% Tris-Tricine pre-cast gels (Bio-Rad Laboratories, 456–3064) and transferred to polyvinylidene difluoride (PVDF) membranes (Bio-Rad, 162–0177). Following a 30 minute incubation at room temperature with 5% blocking milk (Bio-Rad, 170–6404) in 1X tris-buffered saline with tween (TBST), membranes were incubated overnight with primary antibodies for immunodetection of the following proteins: LAMP1 (University of Iowa Hybridoma Bank, H4A3); LC3 (rabbit anti-LC3, Abcam, ab51520); α-syn (Santa Cruz Biotechnology, Inc., SC7011); and APP CTF (Covance Inc, SIG-39152). Membranes were washed with 1X TBST containing 0.1% Tween 20 and then incubated with secondary IgG-HRP conjugated antibody for 1 h at room temperature. After washing blots, SuperSignal West Femto Chemiluminescent Substrate (Thermo Scientific, PI-34096) was used to detect α-syn and Enhanced chemilumescence (ECL; Thermo Scientific, PI-32106) was used to detect LAMP-1, LC3 and APP. Blots were then stripped with Restore Western Blot stripping buffer (Thermo Scientific, 21059) and probed for actin (Sigma, A1978) to normalize for gel loading. Films with detected bands of interest were scanned using Adobe Photoshop and band intensities were calculated using UN-SCAN-IT gel digitizing software (Silk Scientific, Inc.).

### Assessment of Autophagic Flux

Following nucleofection, plating for experiments and 44–46 h following media change, subsets of cells in six well plates were treated with 100 μM chloroquine (Sigma, C6628) plus 200 μM leupeptin (Sigma, L9783) to inhibit lysosome function. At 48 h following initial media change (equaling 2–4 h following treatment with lysosome inhibitors) lysates were processed as above for western blot analysis of LC3-II and actin loading control.

### Assessment of Autophagosome-Lysosome Fusion

Following nucleofection with ATP6V0C siRNA or non-target control siRNA, cells plated in eight well glass chamber slides were treated for 48 h with DMSO and subsequently fixed for 15 min at 4°C using Bouin's fixative (75% saturated picric acid, 23.8% of a 37% w/v formaldehyde solution, 4.7% glacial acetic acid) in the presence of 0.5% saponin (Sigma, 47036). Following incubation for 30 min with 1X PBS blocking buffer containing 1% bovine serum albumin, 0.2% non-fat dry milk and 0.3%Triton X-100, cells were incubated overnight at 4°C in blocking buffer without Triton X-100 and containing rabbit anti-LC3 antibody (Sigma, L7543) to detect autophagosome punctae. Following PBS wash, fixed cells were incubated for 1 h with SuperPicTure (Invitrogen, 879263) anti-rabbit IgG secondary antibody. Following PBS wash, fixed cells were fixed cells were subjected to tyramide signal amplification (incubation for 30 min with Cy3 plus tyramide, Perkin EImer, FP1170) to detect LC3. Next, fixed cells were incubated for 10 min with 3% hydrogen peroxide to neutralize residual peroxidase from the first secondary antibody. After PBS wash and 30 min incubation with blocking buffer, fixed cells were incubated overnight at 4°C in blocking buffer containing the second primary antibody, mouse anti-human LAMP-1 (University of Iowa Hybridoma Bank, H4A3) to detect lysosomes. Following subsequent PBS wash, fixed cells were incubated for 1 h with Vector Impress (Vector Labs, MP-7402) anti-mouse IgG. Following PBS wash, tyramide signal amplification was performed (incubation for 30 min with FITC plus tyramide, Perkin Elmer, FP1168) to label LAMP-1. Following PBS wash, fixed cells were last incubated with bis-benzimide (0.2 μg/ml in PBS, Sigma) to label nuclei. Images were captured using a Zeiss Observer.Z1 laser scanning microscope equipped with a Zeiss 40X Plan-Apochromat objective and imaged using Zen 2008 LSM 710, V5.0 SP1.1 software.

### Assessment of Cell Death Morphology and Neurite Length

After treatment for 48 h with 0–10 nM bafilomycin A1, nucleofected cells plated on eight well glass chamber slides were fixed with Bouin's fixative for 15 min, 4°C. Cell death morphology was visualized via transmitted light and DIC optics on a Zeiss Observer.Z1 laser scanning microscope equipped with a Zeiss 40X 1.3 DIC M27 Plan-Apochromat objective and imaged using Zen 2008 LSM 710, V5.0 SP1.1 software. Neurite length was assessed by first capturing images using Olympus BX51 microscope (Olympus of the Americas) and a 100X UPlan FLN objective. Neurites were measured using the tracing tool within Neurolucida software (MBF Bioscience, version 5.65). Cells identified for measurements had neuritic processes that did not form a synapse with neighboring cells. Data files collected using Neurolucida were quantified using the Nueurolucida Explorer program to determine average neurite length per cell.

### Quantification of Percent Cell Death

Cell death was quantified using propidium iodide (PI; Life Technologies, V13242) staining and flow cytometry. Following treatment for 48 h cells with 0–100 nM bafilomycin A1, nucleofected cells were incubated with PI for 15 min at a final concentration of 1 μg/mL. Cells were then prepared for flow cytometry as above for assessment of acidic vesicle pH and quantified as the percentage of cells that were PI-positive relative to vehicle control using FlowJo software.

### Statistical Analysis and Figure Preparation

Quantitative real time PCR data were expressed as mean ± SEM percent knockdown for ATP6V0C. LTR staining was presented as mean ± SEM fluorescence intensity. Western blot band intensities for LAMP-1, LC3-II, α-syn and APP-CTFS were normalized to actin loading control, and were expressed (mean ± SEM) either as such to assess bafilomycin A1 concentration responsiveness, or further normalized for each ATP6V0C siRNA condition as mean ± SEM fold change relative to its companion non-target control to determine effects of genetic manipulation. Neurite length was presented as mean ± SEM average length (μm) per cell. Cell death was quantified as mean ± SEM percentage of PI-positive cells. To determine effects of genetic manipulation, between groups comparisons of Non-target vs. ATP6V0C siRNA were evaluated for significance using either one-sample t-test (LTR data; quantitative real-time PCR data; western blot data normalized as fold change relative to the companion non-target control) or two-sample t-test (assessment of neurite length and percent cell death). In addition, to assess concentration responsiveness, within group comparisons of absolute (raw) data were evaluated for significance using one-way ANOVA. Significant ANOVAs were followed by post hoc analysis using Bonferroni's Multiple Comparison Test. For all tests, statistical significance was set *a priori* at p<0.05. Graph Pad Prism was used to perform statistical analysis and generate graphs, and Adobe Photoshop was used to assemble figures. A minimum of three independent replicates were performed for each experimental endpoint.

## Results

### Validation of ATP6V0C knockdown

**Figure 1:**
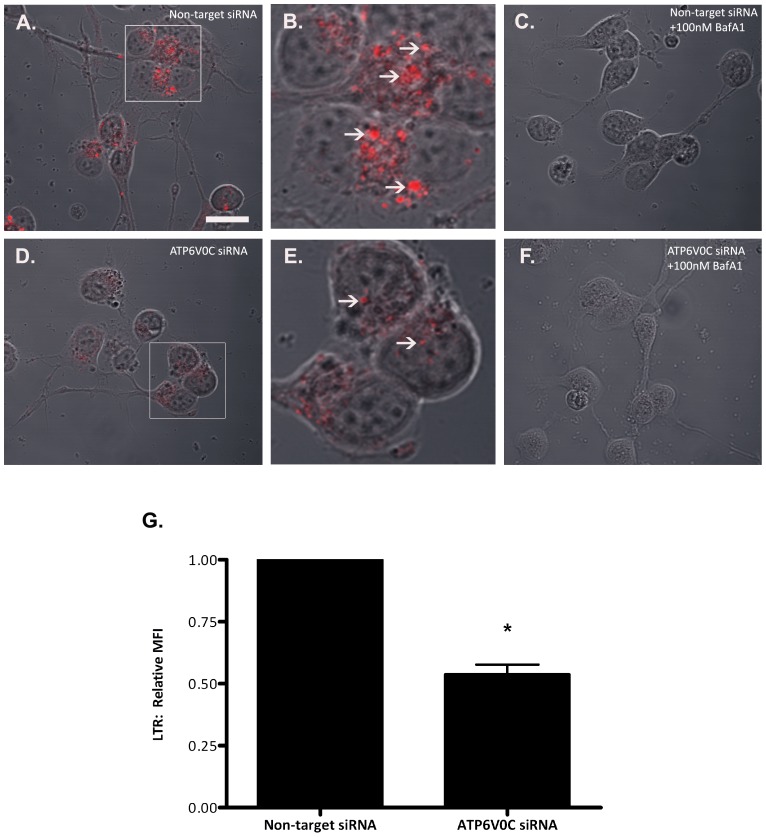
Functional validation of ATP6V0C knockdown. Vesicular acidification following siRNA-mediated knockdown of ATP6V0C was assessed using Lysotracker Red (LTR). LTR-positive punctae were imaged in differentiated SH-SY5Y cells at 96 h following nucleofection of either Non-target siRNA control (A–C) or ATP6V0C siRNA (D–F). DMSO vehicle or 100 nM bafilomycin A1 (BafA1) was added to cells at 3 h prior to the addition of LTR. Scale bar  = 20 μm. Inset boxes in panels A and D are magnified in panels B and E respectively, with arrows indicating LTR-positive punctae. (G) Flow cytometric quantification of LTR relative mean fluorescence intensity (MFI) in vehicle-treated cells at 96 h following nucleofection with Non-target vs. ATP6V0C siRNA. Data are expressed as mean ± SEM and are represented by six independent experiments. ^#^p<0.05 vs. Non-target siRNA using one sample t-test.

We first set out to confirm siRNA-mediated knockdown of ATP6V0C. Quantitative real time PCR analysis was performed on cDNA that was generated from RNA in samples isolated from differentiated SH-SY5Y human neuroblastoma cells harvested 96 h following nucleofection with non-target or ATP6V0C siRNA. Using the comparative cycle threshold (Ct) method (ddCT; [38]) with actin mRNA expression serving as an internal standard, we determined that ATP6V0C mRNA was significantly reduced by 95.01±2.33% (p<0.05 vs. Non-target siRNA using one sample t-test) in ATP6V0C siRNA samples compared to non-target control (Table 1). To determine if ATP6V0C knockdown regulates vesicular acidification as previously reported [34] we performed flow cytometric analysis of LTR staining at 96 h following nucleofection (Fig. 1). Representative images indicated a noticeable reduction in LTR fluorescence following knockdown with ATP6V0C siRNA following vehicle treatment (Fig. 1A, B) compared to non-target control (Fig. 1D, E). As a comparative control, treatment for 3 h with 100 nM bafilomycin A1 appeared to completely ameliorate LTR fluorescence for both non-target control cells and following knockdown with ATP6V0C (Fig. 1C, 1F). Quantification of LTR mean fluorescence intensity in vehicle-treated cells (Fig. 1G) indicated a significant reduction following ATP6V0C knockdown (0.54±0.04) expressed as fold-change relative to non-target control (Fig. 1B), confirming in our knockdown model the importance of ATP6V0C for the maintenance of acidic vesicle pH.

**Table 1:** Expression of ATP6V0C by quantitative real-time PCR.

Sample	Amplicon	Ct^1^	dCt^2^	ddCt^3^	% ATP6V0C expression^4^	% knockdown^5^
ATP6V0C siRNA	ATP6V0C	27.58	4.99	4.32	4.99%	95.01% +/−2.33%*
ATP6V0C siRNA	Actin	22.59				
Non-target siRNA	ATP6V0C	24.29	0.67			
Non-target siRNA	Actin	23.62				

1 Ct is number of cycles to reach threshold, or threshold cycle.

2 dCt  =  average Ct(ATP6V0C) – average Ct(Actin).

3 ddCt  =  dCt(ATP6V0C siRNA) – dCt(Non-target siRNA).

4 % ATP6V0C expression  = 2^–ddCt^.

5 % Knockdown  = 100%–4.99%.

*p<0.05 via one-sample t-test compared to Non-target siRNA control; *n* = 3 replicates.

### ATP6V0C knockdown increases levels of lysosome marker LAMP-1 levels by a low concentration of bafilomycin A1

**Figure 2:**
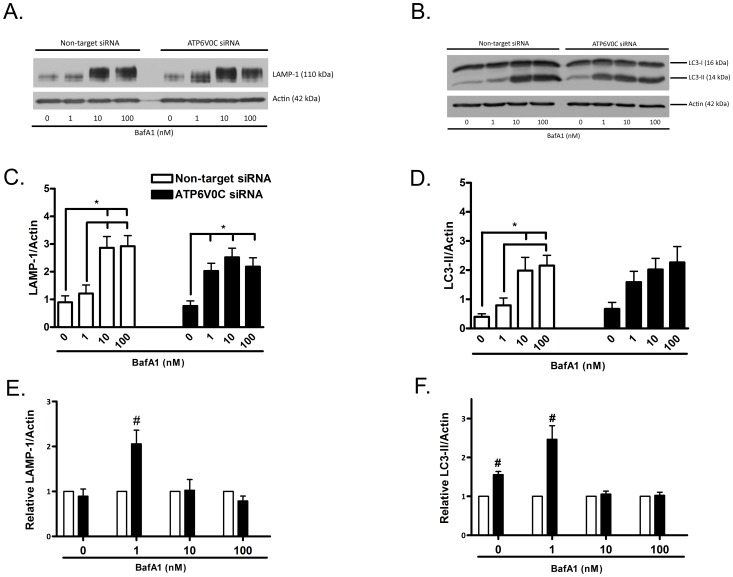
ATP6V0C regulation of autophagy-lysosome pathway markers. Representative western blots for lysosome marker LAMP-1 (A) or autophagosome marker LC3-II (B) from lysates collected following nucleofection with Non-target or ATP6V0C siRNA and subsequent treatment for 48 h with 0–100 nM bafilomycin A1 (BafA1). Blots were stripped and re-probed for actin (42 kDa) to normalize for gel loading. Data from at least six independent experiments are presented graphically in panels C–F. Within group comparisons of BafA1 concentration responsiveness (Non-target siRNA, open columns, left; ATP6V0C siRNA, filled columns, right) were determined for LC3-II (C) or LAMP-1 (D) by expressing mean ± SEM band intensities relative to actin loading control. All lines above columns indicate significant within-group differences with respect to concentration (*p<0.05 using one-way ANOVA and Bonferroni's post-hoc test). Comparisons between groups (Non-target vs. ATP6V0C siRNA) for LC3-II (E) and LAMP-1 (F) were determined for each concentration of BafA1 by expressing mean ± SEM fold changes for each ATP6V0C siRNA condition relative to its companion Non-target control. ^#^p<0.05 vs. corresponding Non-target siRNA control using one-sample t-test.

Levels of LAMP-1 (lysosome-associated membrane protein-1) have been shown previously to increase following lysosomal stress [39]–[41]. To further determine the effects of ATP6V0C knockdown on acidic vesicles we performed western blot analysis to assess LAMP-1 levels at 48 h following treatment (Fig. 2A, C, E). Levels of LAMP-1 were assessed following treatment with vehicle control or following treatment with bafilomycin A1 at concentrations shown previously to have either little effect on inhibiting V-ATPase or vesicular pH (1 nM) or cause profound inhibition of V-ATPase and vesicular acidification at ≥10 nM [6], [8], [42]. In non-target siRNA control cells, significant increases in LAMP-1/actin ratios were observed following treatment with 10 nM (2.89±0.41) and 100 nM (2.92±0.39) bafilomycin A1 compared to treatment with 0 nM (0.90±0.23) or 1 nM (1.22±0.31) concentrations (Fig. 2C, open columns). In ATP6V0C siRNA cells (Fig. 2C, filled columns), the concentration responsiveness of bafilomycin A1 shifted such that significantly higher LAMP-1/actin ratios were observed at all three concentrations tested (1 nM: 2.03±0.28; 10 nM: 2.52±0.33; 100 nM: 2.18±0.32) compared to that observed in 0 nM vehicle control (0.76±0.18). When effects of ATP6V0C siRNA knockdown were compared directly to that of non-target siRNA control (Fig. 2E), a significant 2.06±0.30 fold increase in relative LAMP-1/actin was observed at the 1 nM concentration, whereas fold changes by 0 nM vehicle control (0.89±0.16), 10 nM (1.03±0.24) or 100 nM (0.79±0.11) concentrations of bafilomycin A1 were not significantly different from non-target control. Together these findings indicate that ATP6V0C knockdown shifted the concentration responsiveness of cells to bafilomycin A1 by increasing LAMP-1 levels at a lower concentration not ordinarily associated with lysosome inhibition.

### ATP6V0C knockdown increases basal and stress-induced accumulation of autophagosomes

To determine if ATP6V0C deficiency affected the basal vs. bafilomycin A1-induced accumulation of autophagosomes we performed western blot analysis for LC3-II (Fig. 2B, D, F). Assessing bafilomycin A1 concentration responsiveness in non-target siRNA control cells (Fig. 2D, open columns), treatment with 10 and 100 nM bafilomycin A1 produced significantly greater LC3-II/actin ratios (10 nM: 1.99±0.45; 100 nM: 2.16±0.36) compared to 0 nM vehicle control (0.40±0.10), while LC3-II/actin ratios following treatment with 1 nM bafilomycin A1 (0.79±0.10) were significantly different only in comparison to the 100 nM concentration. While a significant one-way ANOVA was obtained in assessing the bafilomycin A1 concentration responsiveness resulting from ATP6V0C knockdown (Fig. 2D, filled columns), post hoc analysis revealed no significant differences in LC3-II/actin ratios in knockdown cells with respect to any concentration of bafilomycin A1. When effects of ATP6V0C siRNA knockdown were compared directly to that of non-target siRNA control (Fig. 2E), treatment with both 0 nM vehicle control (1.55±0.10) and 1 nM bafilomycin A1 (2.36±0.39) exhibited significantly higher fold changes in relative LC3-II/actin in ATP6V0C knockdown cells compared to non-target siRNA control. Fold change differences in relative LC3-II/actin following ATP6V0C knockdown and treatment with 10 nM bafilomycin A1 (1.08±0.09) or 100 nM bafilomycin A1 (1.01±0.09) were not significantly different relative to non-target siRNA control. These findings indicate that ATP6V0C knockdown not only increased the basal accumulation of autophagosomes but also enhanced their accumulation at a “low” concentration of bafilomycin A1. The lack of significant concentration responsiveness following ATP6V0C knockdown may reflect the observed increase in autophagosomes following 0 nM vehicle and 1 nM bafilomycin A1 treatment.

### ATP6V0C knockdown inhibits autophagic flux

**Figure 3:**
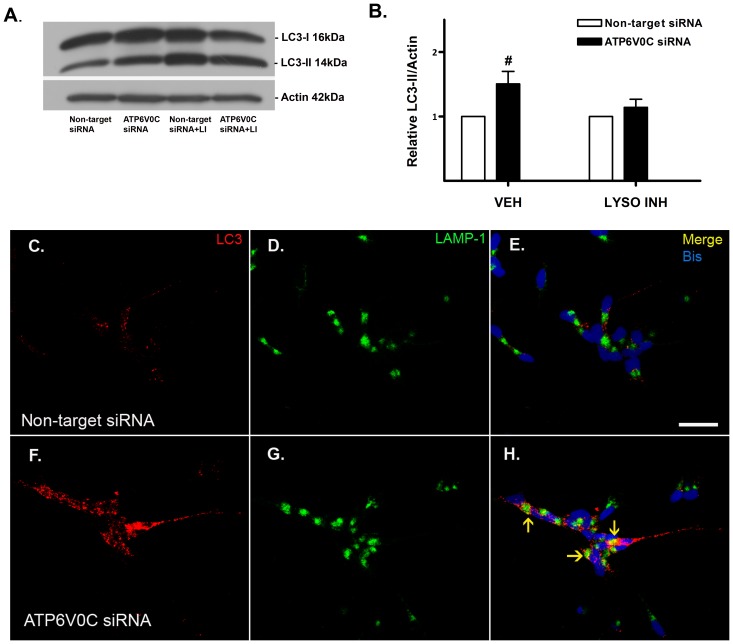
ATP6V0C regulates autophagic flux. Representative western blot for autophagosome marker LC3-II (A) to assess autophagic flux by treating in the presence or absence of the lysosome inhibitors (“LI” in panel A and “LYSO INH” in panel B) chloroquine (100 μM) plus leupeptin (200 μM) for the last 2–4 h of a 48 h time course. Blots were stripped and re-probed for actin (42 kDa) to normalize for gel loading. Data from seven independent experiments are presented graphically in panel B. Comparisons between groups (Non-target vs. ATP6V0C siRNA) were determined by normalizing bands to actin loading control and expressing as mean ± SEM fold change for each ATP6V0C siRNA condition (filled columns) relative to its companion Non-target control (open columns). ^#^p<0.05 vs. corresponding Non-target siRNA control using one-sample t-test. (C–H) Representative confocal microscopy images of fixed cells following nucleofection with either Non-target siRNA control (C–E) or ATP6V0C siRNA (F–H) and subsequent treatment with DMSO vehicle for 48 h. LC3 immunoreactivity (red, C, F) and LAMP-1 (green, D, G) are shown separately and as merged (E, H). Nuclei are stained in panels E and H with bis-benzimide (blue). Co-localization of LC3 and LAMP-1 immunoreactivity (arrows, panels E, H) suggests inhibition of autophagic flux resulting from accumulation of dysfunctional autolysosomes. Scale bar  = 50 μm.

To determine the manner by which knockdown of ATP6V0C caused the accumulation of autophagosomes we assessed autophagic flux by western blot analysis of LC3-II, following inhibition of lysosome function for the last 2–4 h of a 48 h treatment period by treatment with a high concentration of the lysosomotropic agent and weak base chloroquine, and the protease inhibitor leupeptin (Fig. 3A–B) as previously reported [9]. Treatment of non-target siRNA control cells with lysosome inhibitors caused a robust and significant increase in LC3-II/actin (1.39±0.26) compared to vehicle treatment alone (0.69±0.06). When the effects of ATP6V0C siRNA knockdown were compared relative to non-target siRNA control, a significant fold-change increase was observed following vehicle treatment (1.51±0.19), while the fold-change difference in relative LC3-II/actin following treatment with lysosome inhibitors for ATP6V0C siRNA knockdown (1.14±0.13) was not significantly different from non-target control. To further investigate the manner by which autophagic flux was inhibited we performed double-label immunocytochemistry for LC3 and LAMP-1 [9] in vehicle-treated non-target siRNA control cells (Fig. 3C–E) and ATP6V0C siRNA knockdown cells (Fig. 3F–H). Representative images from vehicle-treated non-target siRNA control cells indicate a relative lack of co-localization with LC3 and LAMP-1 punctae (Fig. 3E). In comparison, there were several instances of LC3 and LAMP-1 punctae co-localizing in vehicle treated ATP6V0C knockdown cells (Fig. 3H). Together these results suggest that ATP6C0C knockdown inhibits autophagic flux in a manner caused in part by the accumulation of dysfunctional LC3 and LAMP-1-positive autolysosomes.

### ATP6V0C knockdown induces basal and stressed accumulation of high molecular weight α-syn species

**Figure 4:**
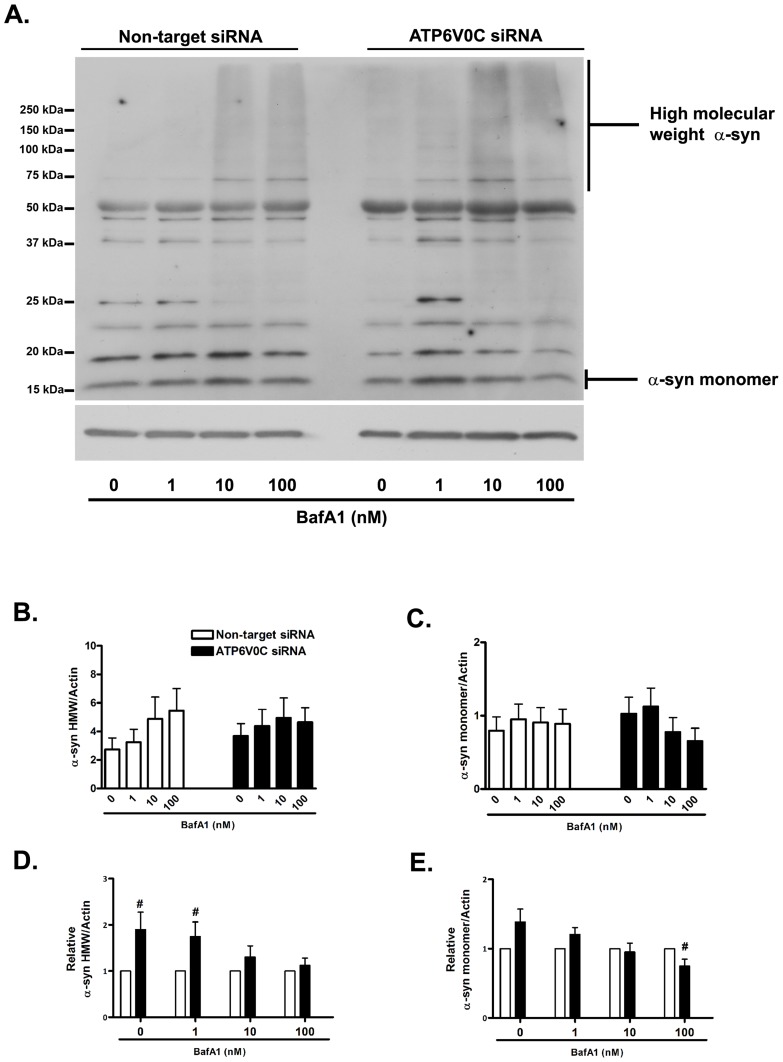
ATP6V0C regulates basal and stress-induced metabolism of alpha synuclein. Representative western blot for α-syn (A) from lysates following nucleofection and subsequent treatment for 48 h with 0–100 nM bafilomycin A1 (BafA1), indicating α-syn high molecular weight (HMW) species (>50 kDa, suggesting multimeric species) and α-syn monomer (∼17 kDa). Blots were stripped and re-probed for actin (42 kDa) to normalize for gel loading. Data from at least six independent experiments are presented graphically in panels B–E. Within groups comparisons of BafA1 concentration responsiveness (Non-target siRNA, open columns, left; ATP6V0C siRNA, filled columns, right) were determined for α-syn HMW species (C) or monomer (D) by expressing mean ± SEM band intensities relative to actin loading control (results of one-way ANOVA were not significant, p>0.05). Comparisons between groups (Non-target vs. ATP6V0C siRNA) for α-syn HMW species (D) or monomer (E) were determined for each concentration of BafA1 by expressing mean ± SEM fold changes for each ATP6V0C siRNA condition relative to its companion Non-target control. ^#^p<0.05 vs. corresponding Non-target siRNA control using one-sample t-test.

It is well-established that lysosome function is important for the effective metabolism of substrates including α-syn that accumulate in age-related neurodegenerative diseases [15], [22], [42]. We have shown previously that treatment of differentiated SH-SY5Y cells with agents that disrupt ALP function causes the accumulation of high molecular weight (MW) α-syn species that are associated with PD pathology [39], [42]._ENREF_17 Specifically, inhibition of V-ATPase with bafilomycin A1 has been shown using western blot analysis to increase levels of α-syn high MW species [25]. Thus western blot analysis was used to determine if knockdown of ATP6V0C altered levels of endogenous high MW α-syn species (defined as greater than 50 kDa) under basal (vehicle) conditions or following treatment with 1–100 nM bafilomycin A1 (Fig. 4). Representative western blot analysis suggests that treatment with bafilomycin A1 induced the accumulation of α-syn high MW species (Fig. 4A). However, one-way ANOVA of pooled data (Fig. 4B) was not significant, thus suggesting a lack of concentration dependence between groups of non-target siRNA control cells treated with 0 nM (2.74±0.81), 1 nM (3.24±0.81), 10 nM (4.98±1.53) or 100 nM (5.46±1.55) bafilomycin A1, or between groups of ATP6V0C siRNA knockdown cells treated with 0 nM (3.68±0.87), 1 nM (4.38±1.17), 10 nM (4.94±1.40) or 100 nM (4.65±1.02) bafilomycin A1. This lack of significant concentration-dependence, at least for non-target siRNA control cells may be explained by the wide rage in absolute values for α-syn/actin ratios observed between different experiments. Regardless, when effects of ATP6V0C siRNA knockdown were compared directly to that of non-target siRNA control (Fig. 4D), treatment with both 0 nM vehicle control (1.90±0.38) and 1 nM bafilomycin A1 (1.75±0.32) exhibited significantly higher fold changes in relative α-syn high MW species/actin compared to non-target siRNA control. Fold changes between observed following treatment with 10 nM (1.31±0.24) or 100 nM (1.12±0.16) bafilomycin A1 were not significantly different. Levels of α-syn monomer (17 kDa) were also assessed in non-target siRNA control and ATP6V0C siRNA knockdown cells (Fig. 4A). Similar to α-syn high MW species (Fig. 4B), one-way ANOVA was not significant, suggesting a lack of bafilomycin A1 concentration responsiveness for ratios of α-syn monomer/actin (Fig. 4C) between groups of non-target siRNA control cells treated with 0 nM (0.79±0.19), 1 nM (0.95±0.21), 10 nM (0.91±0.21) or 100 nM (0.89±0.21) bafilomycin A1, or between groups of ATP6V0C siRNA knockdown cells treated with 0 nM (1.03±0.23), 1 nM (1.12±0.25), 10 nM (0.78±0.20) or 100 nM (0.65±0.18) bafilomycin A1. Interestingly, when the effects of ATP6V0C siRNA knockdown were compared directly to that of non-target siRNA control (Fig. 4E), a significant fold-change decrease in α-syn monomer was observed in ATP6V0C siRNA knockdown cells treated with 100 nM bafilomycin A1 (0.75±0.10), even though concomitant increases in α-syn high MW species were not observed at this concentration. Significant differences in α-syn monomer were not observed in ATP6V0C knockdown cells treated with vehicle control (0 nM: 1.38±0.19) or other concentrations of bafilomycin A1 (1 nM: 1.21±0.10; 10 nM: 0.95±0.13). In summary, while treatment with bafilomycin A1 did not cause concentration-dependent increases in α-syn, our findings suggest that ATP6V0C deficiency alters both the basal and stress-induced metabolism of α-syn.

### ATP6V0C knockdown induces basal and stress-induced accumulation of APP CTFs

**Figure 5:**
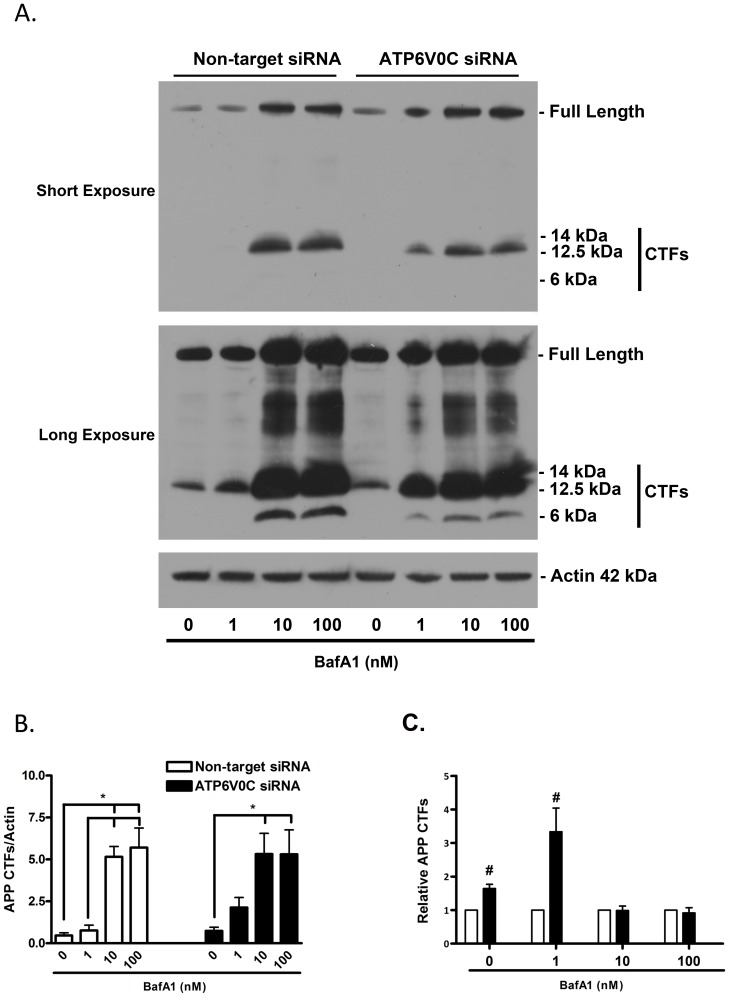
ATP6V0C regulates basal and stress-induced metabolism of APP. Representative western blot (A) for amyloid precursor protein C-terminal fragments (APP CTFs) from nucleofected cells following 48 h treatment with 0–100 nM bafilomycin A1 (BafA1). An antibody was used that recognized both full-length APP (∼110 kDa) and APP CTFs (≤15 kDa). Sizes indicated for CTFs are predicted relative to migration of molecular weight marker and correlate to sizes as previously published (please see results section for further information). In addition to a short-exposure (5 min) blot, a long-exposure (2 h) blot is shown to indicate APP CTFs in vehicle-treated cells. Blots were stripped and re-probed for actin (42 kDa) to normalize for gel loading. APP CTFs from long-exposure blots quantified from four independent experiments are expressed graphically (B–C). Within groups comparisons of BafA1 concentration responsiveness (Non-target siRNA, open columns, left; ATP6V0C siRNA, filled columns, right) were determined (B) by expressing mean ± SEM band intensities relative to actin loading control. All lines above columns indicate significant within-group differences with respect to concentration (*p<0.05 using one-way ANOVA and Bonferroni's post-hoc test). Comparisons between groups (Non-target vs. ATP6V0C siRNA) were determined for each concentration of BafA1 by expressing mean ± SEM fold changes for each ATP6V0C siRNA condition relative to its companion Non-target control. ^#^p<0.05 vs. corresponding Non-target siRNA control using one-sample t-test.

The importance of intact ALP function for the effective degradation of APP and its enzymatic cleavage products, termed C-terminal fragments (CTFs) is also well documented [18], [30], [32]. Treatment with bafilomycin A1 and other inhibitors of lysosome function have been shown previously to promote the accumulation of APP CTFs [30], [32]. To determine if ATP6V0C regulated the basal vs. stressed accumulation of endogenous APP CTFs we performed western blot analysis using an antibody that recognizes both full-length APP and its CTFs (Fig. 5). CTFs were defined in our study as ≤15 kDa based on previously published reports [43] and were quantified as such. APP CTFs observed in our study correspond in size to the C99 fragment (14 kDa), formed by sequential β-and γ-secretase cleavage of APP in the “amyloidogenic” pathway; the C83 fragment (12.5 kDa), formed by β-and α-secretase cleavage of APP in the “non-amyloidogenic” pathway; and the APP intracellular domain (AICD), formed by both amyloidogenic and non-amyloidogenic pathways [30]–[32], [43]. Bafilomycin A1 concentration responsiveness was first assessed for ratios of APP-CTFs/actin (Fig. 5B) in non-target siRNA control cells (open columns) and ATP6V0C siRNA knockdown cells (filled columns). Results of one-way ANOVA and post-hoc analysis revealed significant increases in ratios of APP CTFs/actin following treatment with bafilomycin A1 at 10 (5.15±0.62) and 100 nM (5.71±1.16) concentrations, compared to that of treatment with either 0 nM (0.47±0.16) or 1 nM (0.76±0.32) concentrations. For ATP6V0C siRNA knockdown cells, significant differences in fold-change APP CTFs/actin were observed at 10 nM (5.33±1.23) and 100 nM (5.31±1.45) concentrations of bafilomycin A1 when compared to those in the 0 nM vehicle control group (0.74±0.22) but not following treatment with 1 nM bafilomycin A1 (2.13±0.60). When the effects of ATP6V0C siRNA knockdown were compared directly to non-target siRNA control (Fig. 5C), significant increases in ratios of APP CTFs/actin relative to siRNA control were revealed for both 0 nM (1.64±0.12) and 1 nM (3.33±0.71) concentrations of bafilomycin A1, while comparisons between 10 nM (0.99±0.13) and 100 nM (0.92±0.16) concentrations were not significantly different. These results suggest that ATP6V0C plays a functional role in the effective degradation of APP CTFs, some of which exhibit documented neurotoxic potential.

### ATP6V0C deficiency induced markers of neurotoxicity

**Figure 6:**
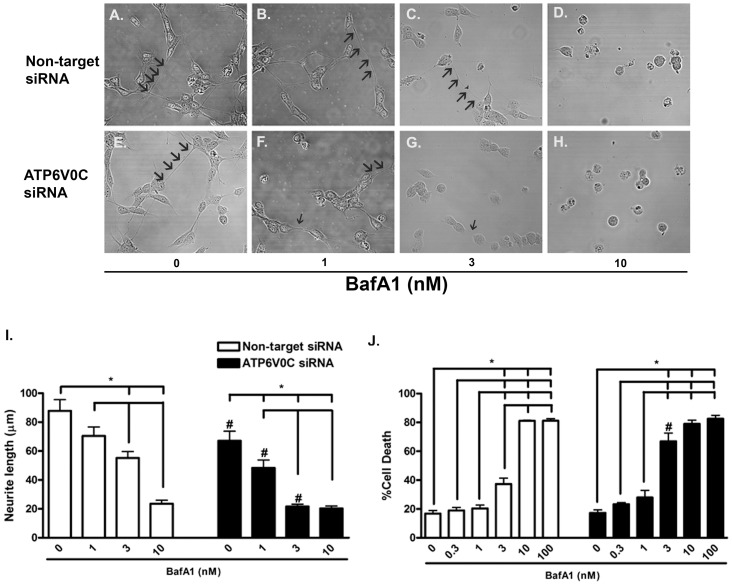
ATP6V0C knockdown exhibit enhanced markers of cytotoxicity. Representative confocal microscopy images obtained from differentiated SH-SY5Y cells following nucleofection with Non-target (A–D) or ATP6V0C (E–H) siRNA and subsequent treatment for 48 h with 0 (A, E), 1 (B, F), 3 (C, G) or 10 (D, H) nM bafilomycin A1 (BafA1). Arrows indicate neuritic processes. Scale bar  = 50 μm. Neurite length (μm) is expressed graphically (I) and represents results (mean ± SEM) from three independent experiments (10 cells per condition in each experiment). Percent cell death (percentage of propidium iodide (PI)-positive cells quantified using flow cytometry) is expressed graphically (J) as mean ± SEM with data obtained from a total of nine independent experiments. All lines above columns indicate significant within-group differences with respect to concentration (*p<0.05 using one-way ANOVA and Bonferroni's post-hoc test). Comparisons between groups (Non-target vs. ATP6V0C siRNA) were determined for each concentration of BafA1 using two-sample t-test (^#^p<0.05).

As ATP6V0C deficiency was shown previously to exacerbate stress-induced cell death [34]–[36] we next determined if knockdown of ATP6V0C was similarly toxic to differentiated SH-SY5Y neuroblastoma cells by investigating different markers of cytotoxicity (Fig. 6). Representative cell morphology is pictured for non-target siRNA cells (Fig. 6A–D) or for ATP6V0C siRNA knockdown cells (Fig. 6E–H). DMSO vehicle-treated non-target control cells (Fig. 6A) and ATP6V0C knockdown cells (Fig. 6E) both exhibited normal appearing cell soma and neurites. Neuritic processes imaged in our study appear similar to those observed previously in SH-SY5Y cells [44], [45]. Non-target control cells treated with 1 nM bafilomycin A1 (Fig. 6B) also appeared morphologically similar to vehicle-treated non-target cells (Fig. 6A), but looked healthier than ATP6V0C knockdown cells treated with 1 nM bafilomycin A1 (Fig. 6F), which exhibited comparatively shorter neurites. Treatment with 3 nM bafilomycin A1 caused a noticeable reduction in neurite length as well as increased pyknosis in both non-target control cells (Fig. 6C) and ATP6V0C knockdown cells (Fig. 6G), although neurite length in ATP6V0C knockdown cells treated with 3 nM bafilomycin A1 appeared shorter than non-target control cells treated at the same concentration. Treatment with 10 nM bafilomycin A1 caused pronounced pyknosis and neurite loss in both non-target control cells (Fig. 6D) and ATP6V0C knockdown cells (Fig. 6H). To quantify our morphological observations we measured neurite length (Fig. 6I). One-way ANOVA was performed followed by post hoc analysis to determine bafilomycin A1 concentration responsiveness for non-target siRNA control-treated cells (Fig. 6I, open columns) or ATP6V0C siRNA knockdown cells (Fig. 6I, filled columns). For non-target siRNA control cells, vehicle control neurite length (87.77±7.80) was significantly greater than that observed at 3 nM (55.22±4.38) or 10 nM (23.58±2.35). Neurite length in non-target control cells following treatment with 10 nM bafilomycin A1 was also significantly less than in cells treated with 1 nM (70.42±6.18) or 3 nM bafilomycin A1. In ATPV0C knockdown cells, neurite length following treatment with DMSO vehicle (67.15±6.54) was significantly greater than that measured following treatment with all other concentrations of bafilomycin A1 (1 nM: 48.38±5.38; 10 nM: 21.61±1.61; 10 nM: 20.29±1.67). In addition, neurite length in 1 nM bafilomycin A1-treated cells was significantly greater than that of 3 and 10 nM concentrations. When neurite lengths in ATP6V0C knockdown cells were compared to those in non-target control cells using two-sample t-test, significant reductions in neurite lengths were observed in ATP6V0C knockdown cells at 0 nM, 1 nM and 3 nM concentrations of bafilomycin A1but not at the 10 nM concentration.

Flow cytometric analysis was also used to quantify the effects of ATP6V0C deficiency on cell death by assessing the percentage of PI-positive cells that accumulated 48 h after treatment with DMSO vehicle or 0.3, 1, 3, 10 or 100 nM bafilomycin A1 (Fig. 6J). Percent cell death (mean ± SEM) was measured as follows for non-target control cells (open columns): DMSO vehicle: 16.75±2.24; 0.3 nM: 18.94±2.09; 1 nM: 20.35±2.36; 3 nM: 37.30±4.10; 10 nM: 81.17±0.36; 100 nM: 81.3±1.48. Following a significant one-way ANOVA and subsequent post hoc analysis, significant increases in percent cell death were observed following treatment with concentrations ≥3 nM bafilomycin A1 compared to treatment with 0 nM vehicle control or 0.3 and 1 nM concentrations. Percent cell death following treatment with bafilomycin A1 was also significantly greater at 10 and 100 nM compared to treatment with 3 nM. Percent cell death for ATP6V0C knockdown cells (filled columns) were as follows: DMSO vehicle: 17.31±2.07; 0.3 nM: 23.30±1.08; 1 nM: 27.97±4.89; 3 nM: 66.98±5.63; 10 nM: 79.07±2.48; 100 nM: 82.57±2.42. Similar to non-target control, results of one-way ANOVA were found to be significant, with post hoc analysis indicating significant increases in percent cell death following treatment with concentrations ≥3 nM bafilomycin A1, compared to treatment with 0 nM vehicle control or 0.3 nM and 1 nM concentrations. However, unlike non-target siRNA-treated cells, percent cell death in 3 nM bafilomycin A1-treated ATP6V0C knockdown cells treated was not different than that observed at 10–100 nM concentrations. Asterisks in Fig. 6J indicate significant differences with respect to concentration of bafilomycin A1. When percent cell death following ATP6V0C knockdown was compared directly to non-target control using two-sample t-test, a significant increase in percent cell death was observed specifically in ATP6V0C knockdown cells treated with 3 nM bafilomycin A1, as indicated by the hash tag (#) in Fig. 6J. Together these findings corroborate our other findings by indicating an increased sensitivity of cells to bafilomycin A1-induced cytotoxicity following knockdown of ATP6V0C.

## Discussion

V-ATPase regulates the proper function of acidic vesicles including endosomes and lysosomes, in part through its maintenance of vesicular pH. This is important not only for the effective transport and delivery of lysosome-associated structural and functional molecules to their pre-destined location, but also for protein degradation pathways that rely on the effective fusion between endosomes, autophagosomes and/or lysosomes. While several studies have indicated the ability of V-ATPase inhibitors including bafilomycin A1 to regulate autophagy-lysosome pathway function and substrate degradation, it is unknown whether the molecular target that bafilomycin A1 binds, the ATP6V0C subunit of V-ATPase, directly mediates these events. Results of the present study show how knockdown of ATP6V0C increases vesicular pH and inhibits basal autophagic flux as well as basal and stress-induced metabolism of autophagy markers/substrates, suggesting that ATP6V0C contributes to the function of the autophagy-lysosome pathway under constitutive conditions and under conditions of stress. In addition, results of our cytotoxicity assays indicate the contribution of ATP6V0C in the maintenance of cell survival under basal and stressed conditions.

Basal vesicular acidification was significantly attenuated following knockdown of ATP6V0C in SH-SY5Y cells differentiated to a neuronal phenotype, which is similar to previous findings in MCF-7 breast cancer tumor cells [34]. This disruption in vesicular acidification is likely responsible for the basal inhibition of autophagic flux observed in our study, which we further characterized by the accumulation of LC3 and LAMP-1 positive punctate co-localizing together that likely represent a population of autolysosomes with a decreased functional capacity for autophagic degradation. Although bafilomycin A1-induced inhibition of V-ATPase has been shown previously to inhibit autophagosome-lysosome fusion [2], and mutants of V_0_ subunits of V-ATPase inhibit vacuole fusion in a pH-independent manner [11], our results suggest that basal knockdown of ATP6V0C in the absence of pharmacological inhibition by V-ATPase inhibition has the capacity to inhibit autophagic flux through its inhibition of lysosomal pH in a manner that does not require fusion block.

While vesicular acidification and autophagic flux were significantly inhibited, and LC3-II levels significantly increased following knockdown of ATP6V0C under basal conditions, we did not observe a corresponding increase in basal LAMP-1 levels. Although we observed an increase in LAMP-1 and LC3 co-localization that we attributed to inhibition of autophagic flux, it is possible that residual ATP6V0C molecules persisting after knockdown allowed for an incomplete inhibition of basal autophagy that would preclude LAMP-1-positive lysosomes from accumulating in size and/or number. Alternatively, it is possible that basal ATP6V0C deficiency disrupts the participation of V-ATPase in a recently identified, complex signaling network involving mTORC1 and TFEB (transcription factor EB) [46], [47]. TFEB is considered a master regulator for the transcription of lysosome-and autophagy associated genes [48]. Thus a disruption in TFEB signaling could also explain why basal LAMP-1 levels were not increased following knockdown of ATP6V0C, a possibility that is worthy of future investigation.

The increase in basal levels of α-syn and APP observed following knockdown suggests a potential contribution of ATP6V0C and V-ATPase to the onset and progression of age-related neurodegenerative disease. To date, alterations in ATP6V0C have yet to be reported in human neurodegenerative disease. A previous study has indicated increased expression of the E3 ubiquitin ligase protein RNF182 in Alzheimer's disease brain [49]. RNF182 was shown using *in vitro* studies to bind ATP6V0C with high affinity and target it for degradation [49], thus suggesting the potential for disruption of ATP6V0C function in Alzheimer's disease. As such, future investigation of ATP6V0C expression patterns in brains of neurodegenerative disease patients is warranted. Even if ATP6V0C expression patterns are found to be unaltered in human neurodegenerative disease, recent studies of ATP6V0C over-expression suggest the potential for its therapeutic benefit. Over-expression of ATP6V0C in mouse substantia nigra was shown recently to attenuate behavioral deficits following treatment with a dopaminergic neurotoxin [50], suggesting its potential utility as a therapeutic target for preventing neurodegeneration. Furthermore, over-expression of ATP6V0C has been shown *in vitro* to induce HIF-1α gene expression in a pH-independent manner [51] providing further proof of principle that over-expression of one subunit of V-ATPase has functional consequence.

In several of our experimental endpoints, knockdown of ATP6V0C consistently and significantly enhanced the sensitivity of cells to low concentrations of bafilomycin A1. Levels of LAMP-1, LC3-II, α-syn and APP CTFs were all significantly higher, and neurite lengths shorter in knockdown cells following treatment with 1 nM bafilomycin A1, a concentration we have shown previously to have little if any influence on autophagy-associated markers or vesicular pH in non-transfected cells [8], and induce protection against chloroquine-induced death and accumulation of autophagic substrates [42]. ATP6V0C knockdown also caused a significant further reduction in neurite length and induction of cell death following treatment with 3 nM bafilomycin A1. The most plausible explanation for these findings is the incomplete nature of ATP6V0C knockdown, with a resultant decrease in residual binding sites causing a shift in bafilomycin A1 concentration responsiveness that would allow lower concentrations of bafilomycin A1 to effectively inhibit V-ATPase and in turn inhibit autophagic degradation and induce cell death. It would be interesting for future studies to determine if knockdown of ATP6V0C prevents the ability of low concentrations of bafilomycin A1 to attenuate ALP-associated dysfunction, as we have shown previously in our laboratory [42].

Although we observed an approximate 95% reduction in ATP6V0C mRNA, it is possible that heightened sensitivity of knockdown cells to bafilomycin A1 could be explained by residual pools of ATP6V0C protein that were translated prior to knockdown. Our laboratory has tried to detect ATP6V0C protein using several commercially available antibodies and to date we have been unable to reliably detect its protein levels, thus limiting our current assessment of ATP6V0C knockdown to its mRNA levels. Alternatively, these results may be explained in part by the existence of different bafilomycin A1 binding sites within V-ATPase, such as ATP6V0A. Isoforms of ATP6V0A have been shown previously to regulate V-ATPase-dependent H^+^ transport [1] and are sensitive in yeast to bafilomycin A1-dependent inhibition [52]. Future study of ATP6V0A could provide meaningful insight with respect to the V-ATPase subunit specificity for bafilomycin A1 and its derivatives for regulating ALP-associated degradation.

While 1 nM bafilomycin A1 did not enhance cell death in ATP6V0C knockdown cells, its ability to shorten neurite length suggest its cytotoxic potential that could have resulted in cell death had measurements been taken at later time points. As bafilomycin A1 inhibits V-ATPase completely at 10 nM [6], [8], it is understandable why treatment of knockdown cells with a ∼three-fold lower concentration of bafilomycin A1 (i.e. 3 nM) shown previously to partially inhibit V-ATPase [6] would be more effective than a ten-fold lower concentration (i.e. 1 nM) in causing neurite shortening and inducing cell death. Timing may also explain why an enhanced knockdown effect was not observed with higher concentrations of bafilomycin A1 (10–100 nM). Had measurements of autophagy substrates and cytotoxicity following treatment with “high” concentrations of bafilomycin A1 been made at time points preceding 48 h, the possibility exists for an unmasking of knockdown-specific effects at these concentrations.

The only knockdown-specific alteration for ATP6V0C observed in the present study following treatment with “high, toxic” concentrations of bafilomycin A1 was a significant decrease in α-syn monomeric species. This result is difficult to interpret, especially considering that α-syn high MW species did not concomitantly increase in knockdown cells following treatment with 100 nM bafilomycin A1. This result may be explained by an enhancement of ubiquitin proteasome system (UPS) function as a consequence to inhibition of the ALP that would in turn enhance degradation of α-syn monomer [15]. In support of this argument, cross-talk and compensation of the ALP or the UPS has been demonstrated recently for the degradation of α-syn following separate inhibition of either pathway [27]. Interestingly, treatment with a cytotoxic concentration of bafilomycin A1 has been shown recently to inhibit the formation of α-syn aggregates [28], which could explain why the bafilomycin A1 concentration dependence for formation of α-syn high MW species in our study was not more robust. However, caution must be used in comparing results between these two studies, as our study assessed levels of endogenous α-syn levels and theirs assessed α-syn aggregation following over-expression [28], as it has been shown recently how relative levels of α-syn can influence the functional capacity of its degradation by the ALP and the UPS [27].

In summary, results of the present knockdown study in neuronal cells indicate a contribution of ATP6V0C towards the basal vs. stress-induced function of the ALP and ALP-associated substrate degradation. Future studies are warranted to validate the utility of ATP6V0C as a therapeutic target to enhance substrate degradation in age-related neurodegenerative disease.
